# Increased triglyceride-glucose index is associated with adverse functional outcome for patients with aneurysmal subarachnoid hemorrhage after surgical clipping and endovascular coiling: insights from a large cohort study

**DOI:** 10.3389/fneur.2025.1622819

**Published:** 2025-09-10

**Authors:** Xielin Tang, Bingcheng Zhu, Minghao Liu, Fa Lin, Runting Li, Wenxiong Song, Jionghao Xue, Liangxue Zhou, Xiaolin Chen

**Affiliations:** ^1^Department of Neurosurgery, West China Hospital, Sichuan University, Chengdu, China; ^2^Department of Neurosurgery, The Affiliated Santai Hospital of North Sichuan Medical College, Mianyang, Sichuan, China; ^3^Department of Neurosurgery, Beijing Tiantan Hospital, Capital Medical University, Beijing, China; ^4^Stroke Center, Beijing Institute for Brain Disorders, Beijing, China

**Keywords:** insulin resistance, aneurysmal subarachnoid hemorrhage, prognosis, cohort study, triglyceride-glucose index

## Abstract

**Background:**

The triglyceride-glucose index (TyG-i) isrecognized as a simple, cost-effective, and valuable surrogate for insulin resistance, and it has been identified to be associated with the prognosis of cardiovascular diseases. However, limited research has been conducted to explore the relationship between TyG-i and clinical outcome of aneurysmal subarachnoid hemorrhage (aSAH). This study aims to elucidate the association between preoperative TyG-i level and the prognosis of aSAH.

**Methods:**

A total of 467 patients with aSAH admitted to Beijing Tiantan Hospital from January 2015 to September 2022 for inclusion in this study. Unfavorable clinical outcome was defined as modified Rankin Scale (mRS) < 3 at 90 days after discharge. TyG-i was calculated using measurements of triglyceride and fasting blood glucose. Additionally, TyG-body mass index (TyG-BMI), a TyG-derived parameter calculated by TyG-i, height, and weight, was also collected. Multivariate logistic regression analysis was performed to explore association between clinical outcome and TyG-i level, as well as its derivative index.

**Results:**

After multivariate adjustment, the increased TyG-i level was associated with high risk of unfavorable clinical outcome (Odds ratio = 3.474, *p* = 0.002). Multivariable-adjusted spline regression model showed a linear relationship between TyG-i and aSAH prognosis (*p* for nonlinear = 0.202). Moreover, adding TyG-i to conventional risk factors significantly improved the risk prediction of poor prognosis (net reclassification index: 40.17%, *p* < 0.001; integrated discrimination index: 3.24%, *p* = 0.005). Multivariate logistic regression analysis demonstrated that there was no significant association between TyG-BMI and clinical outcome of aSAH.

**Conclusion:**

High preoperative TyG-i levels were associated with increased risks of unfavorable clinical outcome, suggesting that TyG-i may be a valuable prognostic marker for patients with aSAH.

**Clinical trial registration:**

https://clinicaltrials.gov/ct2/show/NCT04785976, identifier NCT04785976.

## 1 Background

Aneurysmal subarachnoid hemorrhage (aSAH) is recognized as a life-threatening neurological condition characterized by high mortality and morbidity rates (1). Hughes et al. reported that nearly 500,000 persons experienced aSAH annually, with more than half of these patients residing in low- and middle-income country (2). Hence, identifying an effective and sensitive predictive indicator may significantly enhance the prognosis and alleviate the economic burden faced by aSAH patients.

Previous researches has identified that peripheral blood markers such as hemoglobin, white blood cell, and various inflammation markers, that are associated with the prognosis of aSAH patients (3–6). The triglyceride-glucose index (TyG-i), calculated from peripheral blood glucose and triglyceride, has been recognized as a valuable predictive tool for the prognosis of cardiovascular disease (7–9). Recently, Huang et al. discovered that TyG-i is significantly correlated with all-cause mortality in hemorrhagic stroke patients (10). However, few studies have examined the association between TyG-i and clinical outcome of aSAH patients. This investigation aims to explore the potential correlation between TyG-i and the prognosis of aSAH patients.

## 2 Methods

### 2.1 Study population

The patients’ data were collected from the LongTEAM registry study (Registration No. NCT04785976), a large, single-center, observational cohort conducted at Beijing Tiantan Hospital in China. This cohort included patients information collected from January 2015 to September 2022. Various imaging techniques, such as computed tomography (CT), computed tomography angiography (CTA), digital subtraction angiography (DSA), and lumbar puncture were utilized to diagnose aSAH. The inclusion criteria contained: (1) age ≥ 18 years; (2) patients admitted to hospital from the emergency department; (3) patients with a single aneurysm; (4) patients admitting to hospital within 72 h after aneurysm rupture and receiving treatment within 72 h after admission; (5) patients treated with surgical clipping or endovascular coiling; (6) patients completing 90-days follow-up. The exclusion criteria included: (1) patients with a history of aSAH or other neurosurgical disease; (2) patients with a history of craniotomy or intracranial vascular interventions (3) patients without the data of preoperative peripheral blood glucose and triglyceride; (4) patients with physical disability caused by previous disease; (5) patients with lacking medical records, laboratory, and radiological information. The informed consent was obtained from patients or their guardian before recruitment.

### 2.2 Data collection

The baseline characteristic included age, sex, treatment modality, preoperative clinical status (including World Federation of Neurological Societies (WFNS) grade, modified Fisher scale (mFS), Graeb score, Subarachnoid Hemorrhage Early Brain Edema Score (SEBES), Hunt-Hess score, and Glasgow coma score (GCS)), preoperative symptoms (including loss of consciousness and seizure), and the length from rupture to admission. The radiological information included preoperative intraventricular hemorrhage (IVH) and the max diameter of aneurysm. In-hospital complications, such as postoperative intracranial infection, abnormal liver function, and urinary system infection were also collected. The laboratory examinations, such as triglyceride (TG), fasting blood glucose (FBG) were obtained from patients’ fasting blood in the first 24 h after admission. The TyG-i is calculated as the formula: ln [TG (mg/dl) × FBG (mg/dl)/2] (11). Moreover, TyG-body mass index (TyG-BMI) was also collected. BMI is calculated as weight divided by the square of height (kg/m^2^). TyG-BMI was calculated as the formula: TyG-i × BMI (12).

### 2.3 Outcome evaluation

Patients received follow-up through telephone consultations or outpatient appointments 90 days after discharge. The modified Rankin Scale (mRS), which ranges from 0 (no symptoms) to 6 (death), is a valuable and effective tool for assessing the functional outcome of patients (13). Favorable outcome was defined as mRS < 3 at 90 days after discharge.

### 2.4 Statistical analysis

In this investigation, categorical variables were presented as percentages. Normally distributed numerical variables were expressed as mean ± standard deviation (SD), while skewed distributed variables were indicated as median (25th percentile, 75th percentile). Student’s t test, Mann–Whitney test, and chi-square test were applied to analyze the differences of baseline characteristics between favorable and unfavorable outcome group. The multivariate logistic regression was conducted to evaluate the relationship between TyG-i and clinical outcome. To reduce the impact of confounding factors, we established three models adjusted in multivariate logistic regression. The crude model included age and sex. The minimally adjusted model contained crude model, Grabe score, SEBES, IVH, GCS, WFNS, Hunt Hess score, loss of consciousness, treatment modality, BMI, hypertension, history of heart disease, hyperlipemia, postoperative ventriculomegaly, abnormal liver function, anemia, pneumonia, and deep vein thrombosis (DVT). The fully adjusted model included minimally adjusted model, max diameter of aneurysm, preoperative glucose (Glu), preoperative urea, preoperative estimated glomerular filtration rate (eGFR), preoperative aspartate transaminase (AST), preoperative albumin (ALB), preoperative cholesterol (CHO), preoperative creatine kinase isoenzymes (CKMb), white blood cell (WBC), monocyte (MONO), neutrophil (NEUT) and preoperative hemoglobin (HGB). The restricted cubic spline (RCS) was employed to assess the dose-effect relationship between TyG-i and clinical outcome in patients with aSAH. The knots were determined at the lowest akaike information criterion (AIC) value to enhance the quality of model fitting. The adjusted factors in RCS analysis included age, gender, Grabe, SEBES, IVH, GCS, WFNS, Hunt Hess score, loss of consciousness, treatment modality, hypertension, history of heart disease, hyperlipemia, BMI, postoperative ventriculomegaly, abnormal liver function, anemia, pneumonia, DVT, max diameter of aneurysm, Glu, urea, eGFR, AST, ALB, CHO, CKMb, WBC, MONO, NEUT, and HGB. Net reclassification improvement (NRI) and integrated discrimination improvement (IDI) were utilized to evaluate the enhancement in model performance accomplished by incorporating new markers into conventional model. In this research, ‘TAPS’ model, which contained age, WFNS grade, mFS grade, Grabe score, white blood cell, and surgical clipping, was defined as conventional model (14). NRI and IDI were calculated to evaluate whether adding TyG-i to conventional model could improve the predictive ability of unfavorable clinical outcome. The subgroup analysis was conducted to evaluate the robustness of association between TyG-i and clinical outcome of aSAH. Finally, multivariate logistic regression analysis with 3 adjusted models was applied to explore the association between TyG-BMI and clinical outcome of patients with aSAH.

## 3 Results

### 3.1 Baseline characteristics

**Figure 1:**
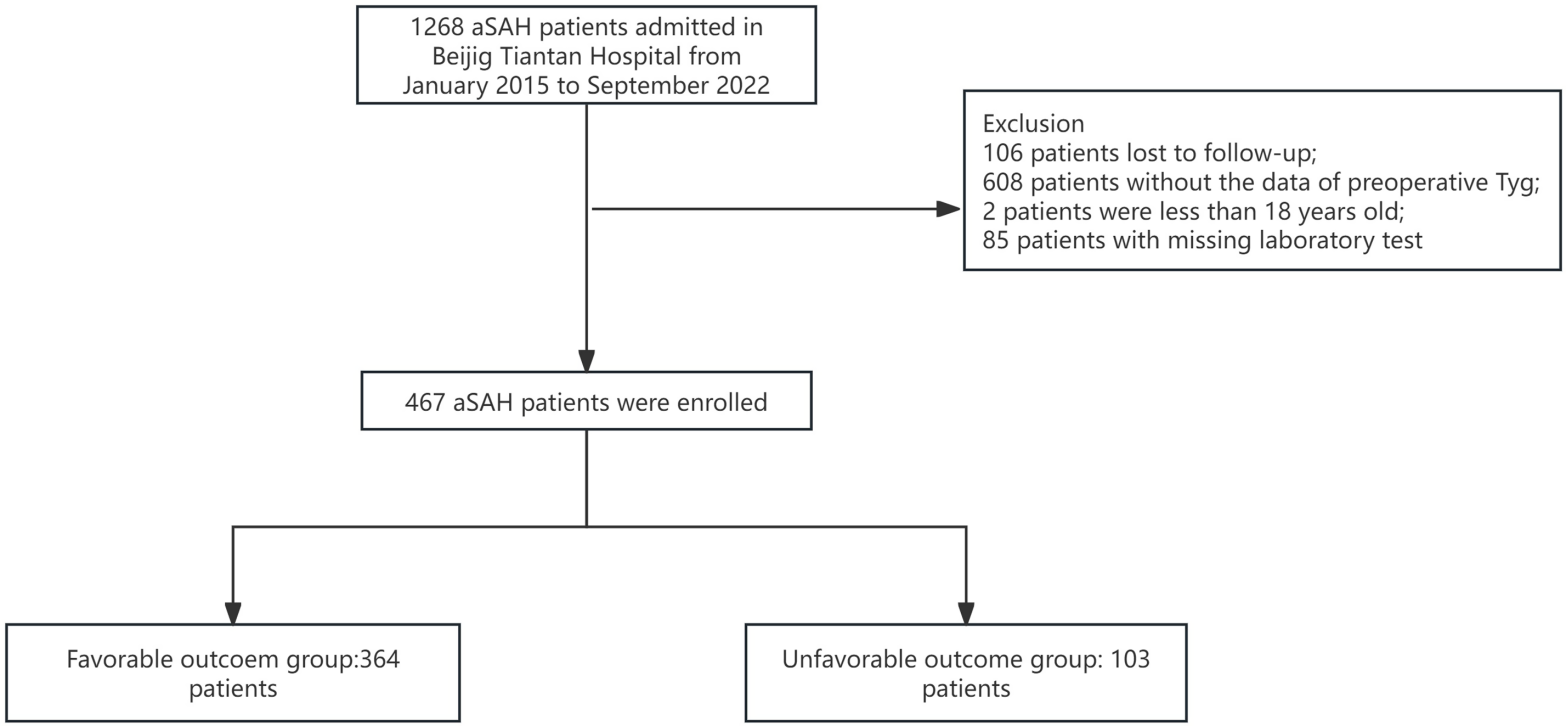
The flow chart of patient enrollment.

**Figure 2:**
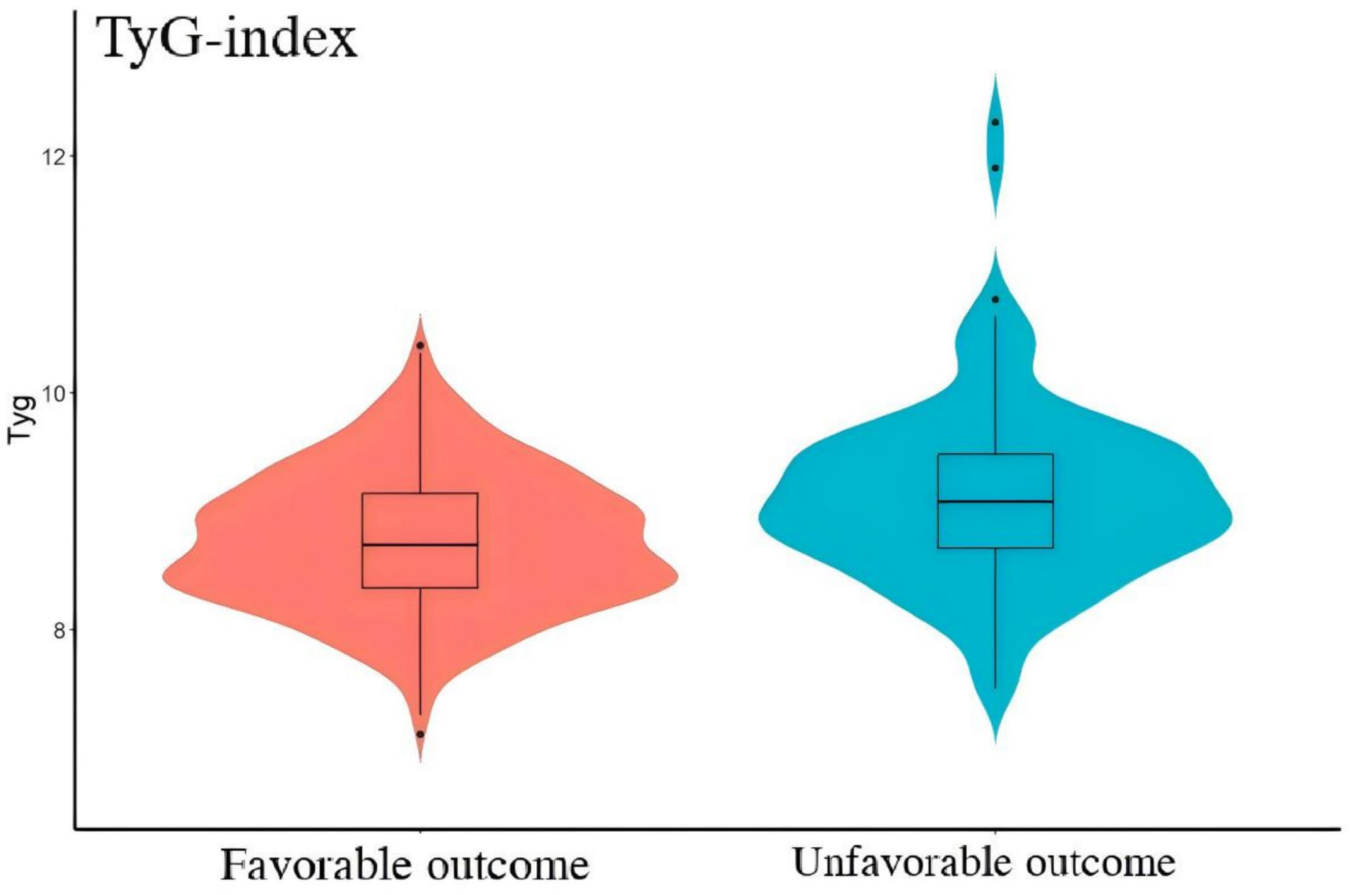
The comparison of TyG-i between good outcome group and unfavorable outcome group. Compared with good outcome group, unfavorable outcome group had a significant higher level of TyG-i (8.72 (8.35–9.15) vs. 9.08 (8.70, 9.48), *p* < 0.001).

All patients were drawn from the LongTEAM registry study. 106 patients were lost to follow-up, 608 patients without the data of preoperative TyG-i, 2 patients were less than 18 years old, and 85 patients with a missing laboratory test. Therefore, a total of 467 patients were enrolled into this study (Figure 1). The comparison between included and excluded patients was presented in Supplementary Table 1. Patients who were excluded from this investigation were more likely to be smokers (17.13% vs. 23.60%, *p* = 0.011), alcohol drinkers (10.92% vs. 21.22%, *p* < 0.001) and with history of heart disease (14.13% vs. 18.85%, *p* = 0.031). The analysis of baseline characteristics was shown in Table 1. 364 patients achieved a favorable clinical outcome while 103 patients experienced an unfavorable outcome. Compared to the favorable outcome group, patients with unfavorable outcome tended to be older (54.00 (47.00–61.00) vs. 61.00 (54.00–69.00), *p* < 0.001), and exhibited a higher score in Grabe (0.00 (0.00–2.00) vs. 2.00 (1.00–3.00), *p* < 0.001), mFS (3.00 (1.00–4.00) vs. 4.00 (3.00–4.00), *p* < 0.001), WFNS (1.00 (1.00–2.00) vs. 4.00 (2.00–5.00), *p* < 0.001), and Hunt-Hess (2.00 (1.50–2.50) vs. 3.00 (4.00–5.00), *p* < 0.001). Furthermore, patients with unfavorable outcome also had a higher prevalence of hypertension (176 (48.35%) vs. 69 (66.99%), *p* < 0.001), hyperlipemia (12 (3.30%) vs. 8 (7.77%), *p* = 0.048), and heart disease (37 (10.16%) vs. 29 (28.15%), *p* < 0.001). Additionally, the incidence of some in-hospital complications such as postoperative ventriculomegaly (31 (8.52%) vs. 20 (19.42%), *p* = 0.002), abnormal liver function (51 (15.01%) vs. 37 (35.92%), *p* < 0.001), anemia (123 (33.79%) vs. 62 (60.79%), *p* < 0.001), pneumonia (112 (30.76%) vs. 80 (77.67%), *p* < 0.001), and DVT (76 (20.87%) vs. 59 (57.28%), *p* < 0.001) were significantly higher among unfavorable outcome group. As for the preoperative laboratory test, Figure 2 presented patients with unfavorable outcome had a higher TyG-i level (8.72 (8.35–9.15) vs. 9.08 (8.70–9.48), *p* < 0.001). There also were significant differences in Glu (7.40 (6.51–8.70) vs. 8.63 (7.30–10.50), *p* < 0.001), eGFR (114.92 (106.33–123.34) vs. 109.57 (100.20–119.45), *p* = 0.001), CO_2_ (22.20 (20.60–23.60) vs. 21.30 (19.50–23.10), *p* = 0.011), AST (20.00 (16.40–25.00) vs. 21.70 (18.00–28.80), *p* = 0.018), CKMb (1.61 (0.97–2.72) vs. 1.98 (1.20–5.31), *p* = 0.004), WBC (12.27 (9.79–14.72) vs. 14.75 (12.10–17.94), *p* < 0.001), MONO (0.37 (0.26–0.51) vs. 0.49 (0.32–0.70), *p* < 0.001), NEUT (10.86 (8.44–13.27) vs. 12.97 (10.46–16.11), *p* < 0.001), and HGB (140.00 (129.00–152.00) vs. 143.00 (134.00–151.00), *p* = 0.044) between unfavorable outcome group and favorable outcome group.

**Table 1:** The comparison of characteristics between good outcome group and unfavorable outcome group.

Characteristics	Outcome		*p*
	Favorable outcome	Unfavorable outcome	
Gender (male:female)	148:216	42:61	0.983
Age, year	54.00 (47.00–61.00)	61.00 (54.00–69.00)	**<0.001**
Treatment modality			**0.007**
Interventional surgery	178	35	
Craniotomy	186	68	
Preoperative evaluation			
Graeb score	0.00 (0.00–2.00)	2.00 (1.00–3.00)	**<0.001**
mFS score	3.00 (1.00–4.00)	4.00 (3.00–4.00)	**<0.001**
SEBES score	2.00 (1.00–4.00)	2.00 (1.00–4.00)	0.812
IVH	200 (54.94)	80 (77.67)	**<0.001**
GCS	15.00 (14.00–15.00)	12.00 (7.00–14.00)	**<0.001**
WFNS grade	1.00 (1.00–2.00)	4.000 (2.00–5.00)	**<0.001**
Hunt Hess score	2.00 (1.00–3.00)	3.00 (2.00–4.00)	**<0.001**
Loss of consciousness, *n* (%)	84 (23.07)	61 (59.223)	**<0.001**
Seizure, *n* (%)	14 (3.846)	7 (6.796)	0.202
The duration of rupture to admission, hours	24.00 (24.00–48.00)	24.00 (17.00–48.00)	0.437
Personal history			
Current smoking, *n* (%)	62 (17.03)	18 (17.48)	0.916
Current drinking, *n* (%)	36 (9.89)	15 (14.56)	0.179
History of ischemic stroke, *n* (%)	18 (4.94)	4 (3.88)	0.653
History of hemorrhagic stroke, *n* (%)	4 (1.10)	1 (0.97)	0.911
History of diabetes, *n* (%)	17 (4.67)	9 (8.74)	0.112
History of hypertension, *n* (%)	176 (48.35)	69 (66.99)	**<0.001**
History of chronic liver disease, *n* (%)	6 (1.65)	3 (2.91)	0.410
History of hyperhomocysteinemia, *n* (%)	22 (6.04)	5 (4.85)	0.648
History of hyperlipemia, *n* (%)	12 (3.30)	8 (7.77)	**0.048**
History of heart disease, *n* (%)	37 (10.16)	29 (28.15)	**<0.001**
History of antiplatelet, *n* (%)	1 (0.27)	1 (0.97)	0.339
History of anticoagulant, *n* (%)	8 (2.20)	5 (4.85)	0.148
Body mass index (kg/m^2^)	24.22 (22.59–26.67)	24.97 (22.36–26.71)	0.718
In-hospital complications			
Postoperative ventriculomegaly, *n* (%)	31 (8.52)	20 (19.42)	**0.002**
Postoperative intracranial infection, *n* (%)	47 (12.91)	19 (18.45)	0.155
Postoperative stress ulcer, *n* (%)	59 (16.21)	27 (26.21)	0.021
Abnormal liver function, *n* (%)	51 (14.01)	37 (35.92)	**<0.001**
Urinary system infection, *n* (%)	10 (2.74)	7 (6.79)	0.053
Anemia, *n* (%)	123 (33.79)	62 (60.19)	**<0.001**
Pneumonia, *n* (%)	112 (30.76)	80 (77.67)	**<0.001**
Disorders of lipoprotein metabolism, *n* (%)	52 (14.28)	19 (18.44)	0.299
DVT, *n* (%)	76 (20.87)	59 (57.28)	**<0.001**
Anterior circulation aneurysm, *n* (%)	36 (9.89)	14 (13.59)	0.283
Max diameter of aneurysm, mm	5.22 (4.00,7.44)	6.000 (4.50,8.40)	**0.021**
Laboratory test			
TyG	8.72 (8.35–9.15)	9.08 (8.70–9.48)	**<0.001**
TyG-BMI	211.74 (193.18–237.81)	225.38 (196.89–244.74)	**0.028**
Preoperative Glu, mmol/L	7.40 (6.51–8.70)	8.63 (7.30–10.50)	**<0.001**
Preoperative Urea, mmol/L	4.50 (3.70–5.40)	4.80 (3.90–5.50)	0.313
Preoperative Cr, μmol/L	55.00 (46.60–66.20)	53.60 (44.90–65.10)	0.397
Preoperative eGFR, ml/min	114.92 (106.33–123.34)	109.57 (100.20–119.45)	**0.001**
Preoperative CO_2_, mmol/L	22.20 (20.60–23.60)	21.30 (19.50–23.10)	**0.011**
Preoperative ALT, U/L	17.40 (13.00–24.90)	18.40 (13.70–25.90)	0.339
Preoperative AST, U/L	20.00 (16.40–25.00)	21.70 (18.00–28.80)	**0.018**
Preoperative TP, g/L	72.50 (69.10–76.10)	73.00 (69.00–76.80)	0.507
Preoperative ALB, g/L	42.80 (40.40–44.90)	42.40 (40.90–45.70)	0.696
Preoperative GLB, g/L	29.70 (27.20–32.80)	30.00 (26.90–32.80)	0.655
Preoperative CHO, mmol/L	4.62 (4.08–5.32)	4.67 (4.08–5.45)	0.274
Preoperative CKMb, ng/ml	1.61 (0.97–2.72)	1.98 (1.20–5.31)	**0.004**
Preoperative WBC, 10^9^/L	12.27 (9.79–14.72)	14.75 (12.10–17.94)	**<0.001**
Preoperative LY, 10^9^/L	0.92 (0.66–1.27)	0.88 (0.72–1.22)	0.924
Preoperative MONO, 10^9^/L	0.37 (0.26–0.51)	0.49 (0.32–0.70)	**<0.001**
Preoperative NEUT, 10^9^/L	10.86 (8.44–13.27)	12.97 (10.46–16.11)	**<0.001**
Preoperative EO, 10^9^/L	0.01 (0.00–0.01)	0.00 (0.00–0.02)	0.656
Preoperative RBC, 10^9^/L	4.51 (4.20–4.83)	4.55 (4.30–4.81)	0.467
Preoperative HGB, g/L	140.00 (129.00–152.00)	143.00 (134.00–151.00)	**0.044**

mFS, modified Fisher scale; SEBES, Subarachnoid Hemorrhage Early Brain Edema Score; IVH, intraventricular hemorrhage; GCS, Glasgow coma score; WFNS, World Federation of Neurological Societies; DVT, deep vein thrombosis; TyG, Triglyceride-glucose; Glu, glucose; Cr, creatinine; eGFR, estimated glomerular filtration rate; ALT, alanine transaminase; AST, aspartate transaminase; TP, total protein; ALB, albumin; GLB, globulin; CHO, cholesterol; CKMb, creatine kinase isoenzymes; WBC, white blood cell; LY, lymphocyte; MONO, monocyte; NEUT, neutrophil; EO, eosinophil; RBC, red blood cell; HGB, hemoglobin. Bold values: *p* < 0.05.

### 3.2 TyG-i levels and clinical outcome

**Figure 3:**
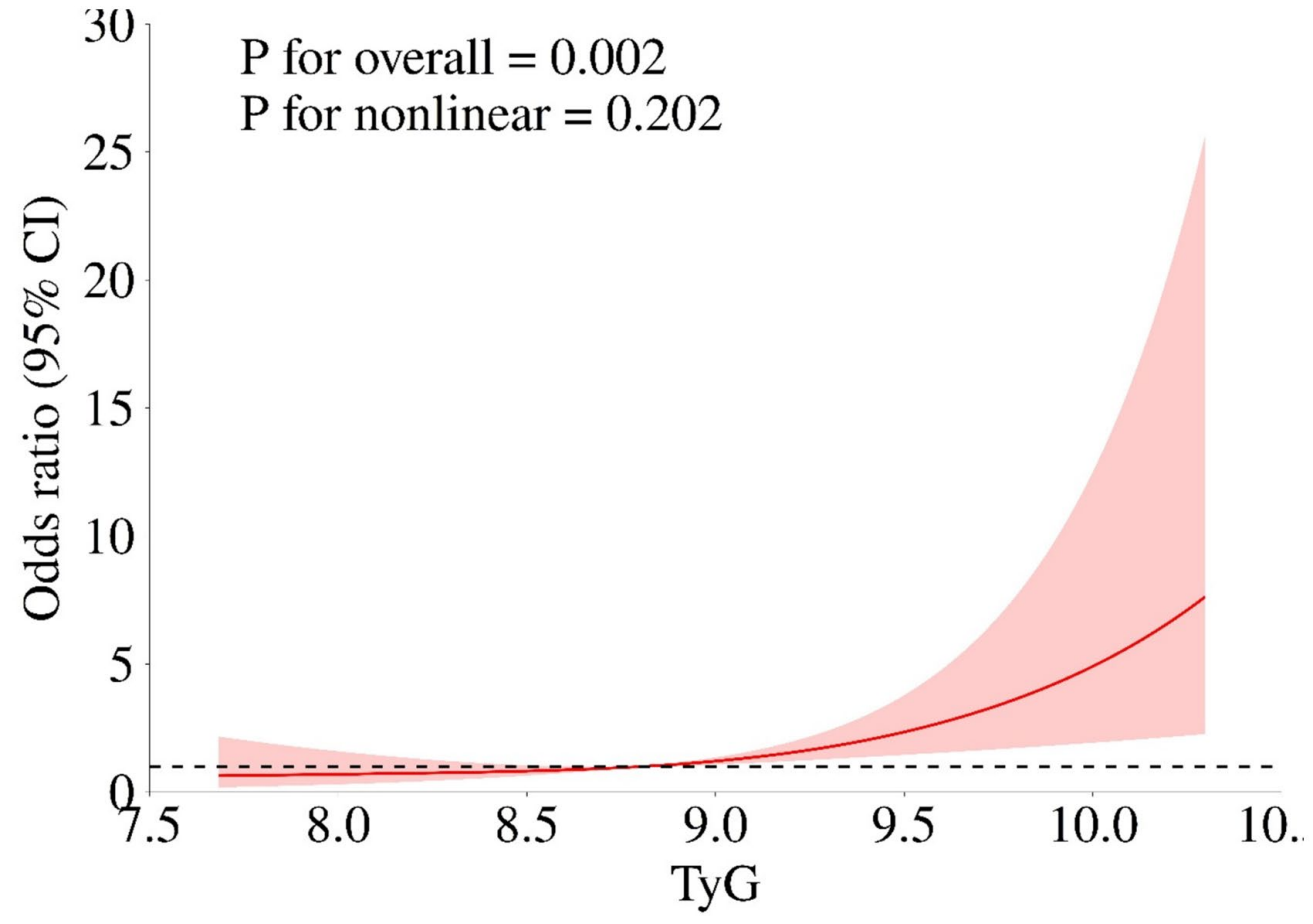
The association of TyG-i index and unfavorable outcome. Age, gender, Grabe, SEBES, IVH, GCS, WFNS, Hunt Hess score, loss of consciousness, treatment modality, hypertension, history of heart disease, hyperlipemia, body mass index, postoperative ventriculomegaly, abnormal liver function, anemia, pneumonia, DVT, max diameter of aneurysm, preoperative Glu, preoperative urea, preoperative eGFR, preoperative AST, preoperative ALB, preoperative CHO, preoperative CKMb, preoperative WBC, preoperative MONO, preoperative NEUT, and preoperative HGB.

Table 2 illustrated the association between TyG-i levels and the clinical outcome of aSAH patients. In fully adjusted model, the risk of unfavorable outcome increased with each increment in TyG-i (Odds ratio (OR): 3.474 (1.536–7.855), 95% Confidence Interval (CI): 1.536–7.855, *p* = 0.002). When TyG-i was evaluated in quartiles based on the distribution, compared with quartile 1 (TyG-i < 8.376), the fully adjusted OR was 6.777 and the 95% CI was 1.455–31.557 (*p* = 0.015). Moreover, multivariable-adjusted RCS was presented in Figure 3. This figure illustrated that the TyG-i had a linear relationship with unfavorable outcome (*p* = 0.002, *p* for non-linearity = 0.202).

**Table 2:** The association between baseline Tyg level and the risk of unfavorable outcome.

	The number of events (unfavorable outcome), *n* (%)	Crude model		Minimally adjusted model		Fully adjusted model	
		OR (95% CI)	*p*	OR (95% CI)	*p*	OR (95% CI)	*p*
All patients	103	2.690 (1.832–3.950)	<0.001	3.445 (1.861–6.376)	<0.001	3.474 (1.536–7.855)	0.002
Tyg tertiles							
Q1 (<8.376)	13	1.0 (Ref)		1.0 (Ref)		1.0 (Ref)	
Q2 (8.376–8.804)	22	1.984 (1.037–3.797)	0.039	2.589 (0.675–9.924)	0.165	2.125 (0.916–4.930)	0.265
Q3 (>8.804–9.268)	28	4.045 (2.169–7.543)	<0.001	4.714 (1.396–15.924)	0.013	4.529 (1.076–19.069)	0.039
Q4 (>9.268)	40			6.860 (2.022–23.281)	0.002	6.777 (1.455–31.557)	0.015

Crude model: age, gender.

Minimally adjusted model: age, gender, Grabe, SEBES, IVH, GCS, WFNS, Hunt Hess score, loss of consciousness, treatment modality, hypertension, history of heart disease, hyperlipemia, body mass index, postoperative ventriculomegaly, abnormal liver function, anemia, pneumonia, and DVT.

Fully adjusted model: age, gender, Grabe, SEBES, IVH, GCS, WFNS, Hunt Hess score, loss of consciousness, treatment modality, hypertension, history of heart disease, hyperlipemia, body mass index, postoperative ventriculomegaly, abnormal liver function, anemia, pneumonia, DVT, max diameter of aneurysm, preoperative Glu, preoperative urea, preoperative eGFR, preoperative AST, preoperative ALB, preoperative CHO, preoperative CKMb, preoperative WBC, preoperative MONO, preoperative NEUT, and preoperative HGB.

### 3.3 Incremental prognostic value of the TyG-i

As presented in Table 3, adding the TyG-i to ‘TAPS’ model improved the risk reclassification for unfavorable outcome (NRI: 40.17%, *p* < 0.001; IDI: 3.24%, *p* = 0.005).

**Table 3:** Reclassification and discrimination statistics for unfavorable outcome of aSAH by Tyg at baseline.

	Continuous NRI, %		IDI, %	
	Estimate (95% CI)	*p* value	Estimate (95% CI)	*p* value
SAH				
Conventional model				
Conventional model + Tyg (continuous)	40.17 (18.75–60.59)	**<0.001**	3.24 (0.95–5.52)	**0.005**

Bold values: *p* < 0.05.

### 3.4 The subgroup analysis of TyG-i

A subgroup analysis was further performed to evaluate the potential modified effect of predetermined factors on the relationship between preoperative TyG-i and clinical outcome. Based on the quartiles of TyG-i, patients were categorized into two groups: a high TyG-i group, defined as those with TyG-i values greater than 8.804, and a low TyG-i group, comprising patients with TyG-i values of 8.804 or less. As shown in Table 4, the results of the subgroup analysis indicated that there were no significant interactions between TyG-i and the specified factors. In fact, all *p*-values for interaction were greater than 0.05, suggesting that the prespecified factors did not significantly influence the relationship between TyG-i and clinical outcomes in this analysis.

**Table 4:** The subgroup analysis for clinical outcome of aSAH patients.

Variables	*n* (%)	TyG-i > 8.804	TyG-i ≤ 8.804	OR (95%CI)	*p*	*p* for interaction
All patients	467 (100.00)	165/233	199/234	2.34 (1.48–3.70)	**<0.001**	
Gender						0.113
Female	277 (59.31)	96/140	120/137	3.24 (1.74–6.02)	**<0.001**	
Male	190 (40.69)	69/93	79/97	1.53 (0.76–3.05)	0.230	
Age older than 65 years						0.618
No	360 (77.09)	139/181	158/179	2.27 (1.28–4.03)	**0.005**	
Yes	107 (22.91)	26/52	41/55	2.93 (1.30–6.61)	**0.010**	
Graeb score						0.406
0–4	433 (92.72)	156/210	191/223	2.07 (1.27–3.36)	**0.003**	
5–12	34 (7.28)	9/23	8/11	4.15 (0.86–19.92)	0.076	
SEBES score						0.896
0–2	249 (53.32)	88/127	103/122	2.40 (1.30–4.46)	**0.005**	
3–4	218 (46.68)	77/106	96/112	2.26 (1.14–4.46)	**0.019**	
mFS Score						0.421
0–2	169 (36.19)	62/76	82/93	1.68 (0.72–3.96)	0.233	
3–4	298 (63.81)	103/157	117/141	2.56 (1.48–4.43)	**<0.001**	
WFNS score						0.225
1–3	361 (77.30)	141/174	174/187	3.13 (1.59–6.18)	**<0.001**	
4–5	106 (22.70)	24/59	25/47	1.66 (0.76–3.59)	0.200	
Hunt Hess score						0.156
1–3	420 (89.94)	158/207	193/213	2.99 (1.71–5.24)	**<0.001**	
4–5	47 (10.06)	7/26	6/21	1.09 (0.30–3.92)	0.900	
IVH						0.860
No	187 (40.04)	70/84	94/103	2.09 (0.86–5.10)	0.106	
Yes	280 (59.96)	95/149	105/131	2.30 (1.33–3.95)	**0.003**	
Loss of consciousness						0.187
No	322 (68.95)	123/153	157/169	3.19 (1.57–6.49)	**0.001**	
Yes	145 (31.05)	42/80	42/65	1.65 (0.84–3.23)	0.143	
Treatment modality						0.714
Endovascular intervention	213 (45.61)	85/110	93/103	2.74 (1.24–6.03)	**0.013**	
Surgical clipping	254 (54.39)	80/123	106/131	2.28 (1.29–4.04)	**0.005**	
Current smoking						0.270
No	387 (82.87)	133/186	169/201	2.10 (1.28–3.45)	**0.003**	
Yes	80 (17.13)	32/47	30/33	4.69 (1.23–17.82)	**0.023**	
Current drinking						0.786
No	416 (89.08)	147/204	181/212	2.26 (1.39–3.69)	**0.001**	
Yes	51 (10.92)	18/29	18/22	2.75 (0.74–10.27)	0.132	
Diabetes						0.376
No	441 (94.43)	154/216	193/225	2.43 (1.51–3.91)	**<0.001**	
Yes	26 (5.57)	11/17	6/9	1.09 (0.20–6.01)	0.920	
Hypertension						0.472
No	222 (47.54)	74/92	114/130	1.73 (0.83–3.61)	0.142	
Yes	245 (52.46)	91/141	85/104	2.46 (1.34–4.50)	**0.004**	
History of heart disease						0.886
No	401 (85.87)	146/194	181/207	2.29 (1.35–3.87)	**0.002**	
Yes	66 (14.13)	19/39	18/27	2.11 (0.76–5.82)	0.151	
History of anticoagulant						0.694
No	454 (97.22)	161/226	195/228	2.39 (1.49–3.81)	**<0.001**	
Yes	13 (2.78)	4/7	4/6	1.50 (0.16–14.42)	0.725	

OR, Odds Ratio, CI, Confidence Interval, mFS, modified Fisher scale; SEBES, Subarachnoid Hemorrhage Early Brain Edema Score; IVH, intraventricular hemorrhage; GCS, Glasgow coma score; WFNS, World Federation of Neurological Societies. Bold values: *p* < 0.05.

### 3.5 Association between TyG-BMI and clinical outcome of aSAH

Baseline characteristics analysis demonstrated that patients in unfavorable outcome group had higher levels of TyG-BMI (211.74 (193.18, 237.81) vs. 225.38 (196.89, 244.74), *p* = 0.028). Multivariate analysis was shown in Supplementary Table 2. After adjusting for all potential covariates, logistic regression analysis demonstrated that there was no significant association between TyG-BMI and clinical outcome of aSAH patients (OR (95%CI): 1.010 (0.998–1.023), *p* = 0.088).

## 4 Discussion

Aneurysmal subarachnoid hemorrhage is widely recognized as a life-threatening disease with poor prognosis and high mortality rates (15). Therefore, enhancing the prognosis of aSAH prognosis is crucial, as it can alleviate the economic burden on both patients and society. Several studies have been conducted to identify risk factors associated with poor aSAH outcome. The Hunt-Hess grade, surgical method, and in-hospital complications have been identified to be associated with poor clinical outcome of aSAH patients (16–18). Recently, the importance of biomarkers in predicting aSAH prognosis has gained increasing attention. In 2019, Ding et al. identified serum neuroglobin as a potential predictor of poor aSAH outcome (19). Several peripheral blood inflammation and nutritional markers, such as neutrophil-to-lymphocyte ratio, systemic immune inflammation index, systemic inflammation response index, HGB, and prognostic nutritional index have been demonstrated to be risk factors of poor prognosis in aSAH patients. Moreover, Pesaresi et al. found the dynamic changes of cerebral spinal fluid (CSF) biomarkers might provide a more valuable insights into the risk identification of aSAH poor prognosis (20). However, few investigations have researched the relationship between metabolic markers and aSAH prognosis.

TyG-i, a newly emerging biochemical index calculated from FBG and fasting TG, has been identified as a potential indicator of insulin resistance (IR) (21). Compared to traditional assessment tools such as homoeostasis model assessment of IR (HOMA-IR) or the hyperinsulinemia-euglycemic clamp test, TyG-i is a more reliable, easily available, and straightforward surrogate for IR. Previous studies have identified that IR plays an important role in the development of heart disease and deterioration of renal function (22). Consequently, TyG-i was initially recognized as predictive factor for prognosis of cardiovascular disease or renal disease. Ji et al. found high TyG-I levels was associated with the occurrence of acute kidney injury and poor renal function in heart failure (HR) patients (7). In 2019, Park et al. demonstrated that TyG-i might promote the development of coronary artery calcification (23). Several researches also showed that TyG-i may be a critical prognosis predictive factor in arterial stiffness, in-stent restenosis and acute coronary syndrome (24). In recent years, more and more studies began to explore the correction between TyG-i and cerebrovascular disease. In 2023, a meta-analysis revealed TyG-i might influence the functional outcome and recurrence rate in ischemic stroke patients (25). However, limited studies were performed to explore the association between TyG-i and prognosis of aSAH.

The aim of this research is to explore the correlation between TyG-i and aSAH prognosis. Based on LongTEAM registry study (Registration No. NCT04785976), we found patients with unfavorable outcome tended to have higher levels of preoperative TyG-i. After adjusting for all potential confounding factors, multivariate logistic analysis illustrated preoperative TyG-i was significantly associated with aSAH prognosis. Yin et al. revealed TyG-i correlated with all-cause mortality of critical ill hemorrhagic stroke (including intracranial hemorrhage and SAH) (10). Xie et al. conducted a retrospective study recruiting 134 patients with SAH and identified higher TyG-i might be associated with poor clinical outcome (26). Hou et al. found elevated TyG-i may increase the risk of poor functional outcome of aSAH (27). These findings are in accordance with the results of our research. Moreover, compared with previous studies, the present study systematically collected data on in-hospital complications and incorporated these complications into the adjusted model. This approach might effectively diminishes the influence of confounding factors when investigating the association between the TyG-i and clinical outcome in patients with aSAH. Additionally, another TyG related index, TyG-BMI was analyzed in this investigation as well.

There are some potential theories for the underlying mechanism behind the association of IR and aSAH prognosis. EBI is an important pathophysiologic process following aSAH, which is able to influence the prognosis significantly (28). According to Zipfel et al., EBI can be classified into two stages: primary injury and secondary injury (1). After aneurysm rupture, the primary injury begins immediately. The hemorrhagic blood extravasates into subarachnoid areas, ventricles, and parenchyma, causing a rapid rise of intracranial pressure (ICP) (29). Meanwhile, blood and hemoglobin breakdown products extravasates into brain and induces secondary injury (30). The secondary injury includes brain edema, microcirculatory dysfunction, blood–brain-barrier disruption, neuroinflammation, and oxidative cascades (31–35). Based on the findings of previous studies, we considered IR might promote the development of several pathological processes among primary and secondary injury. On one hand, IR was considered to be correlated with platelet dysfunction and endothelial cell-dependent vasodilation, like vascular cell adhesion molecule-1 and E-selectin. These proteins were able to elevate permeability of the vascular endothelia, which might increase hemorrhage volume and aggravate the primary injury (36). On the other hand, researchers discovered that IR prevented glucose from entering into neurons for oxidative phosphorylation and inhibited polarization of macrophages, potentially inducing the development of inflammation during acute phase of aSAH, which might increase the risk of unfavorable clinical outcome (37, 38). Alongside the inflammation, IR is also reported to be associated with oxidative stress in brain tissue. Chabowski et al. summarized that IR increased free fatty acids and promoted glucotoxicity, resulting in the overproduction of reactive oxygen species (ROS) (39). In aSAH, excessive ROS production breaks the balance of oxidant and antioxidant composition, causing oxidative stress and exacerbating brain injury (1). Subsequently, a higher TyG-i reflects a more severe IR status, which aggravates the development of EBI and leads to a poor clinical outcome.

Notably, we also analyzed the association between prognosis and TyG-BMI. After adjusting for all confounding factors, no significant correlation was observed between TyG-BMI and the prognosis of aSAH. This phenomenon might be explained by the ‘obesity paradox’ (40). Although obesity is generally considered detrimental in most diseases, several studies suggest it may play a protective role in aSAH patients (40). BMI is an objective indicator of obesity. Rinaldo et al. found high BMI might decrease the risk of unfavorable function outcome for aSAH patients treated with surgical clipping (41). In another retrospective study, elevated BMI was identified to decrease the risk of delayed infarction (42). Hence, as a combination of TyG-i and BMI, the relationship between TyG-BMI and aSAH prognosis still requires further validation. This finding reveals that compared with TyG-BMI, TyG-i not only demonstrates greater accessibility but also exhibits a more definitive correlation with prognosis of aSAH, suggesting its superior suitability for clinical application.

There are some limitations in our study. First, this was single-center research, which might induce potential bias. Second, due to the lack of data, such as preoperative FBG and TG, we excluded a large number of patients. The small sample size limited the subgroup analysis and future research. Third, some parameters, such as diet information, metabolic syndrome, diabetes management, and ongoing pharmacological treatments were not collected. These data could influence TyG-i and might be potential confounding factors. Fourth, TyG-i was only measured in acute phase (0–3 days after aneurysm ruptures). TyG-i measured in different stages are failed to be obtained, which limited in-depth analysis. Additionally, except TyG-i and TyG-BMI, other IR markers are also needed to be analyzed to find more suitable marker for prognostic predictive. Fifth, the dynamic changes of TyG-i during hospitalization are lost. In 2025, Pesaresi et al. highlighted the importance of monitoring CSF biomarkers over time for patients with aSAH (20). Hence, future investigations are still required to confirm the prognostic value of longitudinal monitoring TyG-i in patients with aSAH. Finally, we only applied ‘TAPS’ model to analyze whether TyG-i can increase model predictive ability. According to Hao et al., there were 6 exiting models to predict the prognosis of aSAH (14). Due to the included factors, enrollment criteria, and research endpoint, we failed to perform deeper analysis into the other 5 models. We confirm that a multi-center, prospective study with a large sample and adequate parameters is needed to strengthen our conclusion.

## 5 Conclusion

Our research identified that TyG-i could be considered as a potential prognostic indicator for patients with aSAH. Monitoring TyG-i may be beneficial for aSAH patients. A large randomized controlled trial is needed to identify whether management of TyG-i can improve clinical outcome.

## Data Availability

The original contributions presented in the study are included in the article/Supplementary material, further inquiries can be directed to the corresponding author.

## Glossary

TyG-i: Triglyceride-glucose index
aSAH: Aneurysmal subarachnoid hemorrhage
EBI: Early brain injury
CT: Computed tomography
CTA: Computed tomography angiography
DSA: digital subtraction angiography
WFNS: World Federation of Neurological Societies
mFS: Modified Fisher scale
SEBES: Subarachnoid Hemorrhage Early Brain Edema Score
GCS: Glasgow coma score
IVH: Intraventricular hemorrhage
TG: Triglyceride
FBG: Fasting blood glucose
mRS: Modified Rankin Scale
DVT: Deep vein thrombosis
GLU: Glucose
eGFR: Estimated glomerular filtration rate
AST: Aspartate transaminase
ALB: Albumin
CHO: cholesterol
CKMb: Creatine kinase isoenzymes
HGB: hemoglobin
RCS: Restricted cubic spline
NRI: Net reclassification improvement
IDI: Integrated discrimination improvement
NLR: Neutrophil-lymphocyte ratio
IR: Insulin resistance
HOMA-IR: Homoeostasis model assessment of IR
SAH: Subarachnoid hemorrhage
ICP: Intracranial pressure
ROS: Reactive oxygen specie
