# Supplementary material for: Increased triglyceride-glucose index is associated with adverse functional outcome for patients with aneurysmal subarachnoid hemorrhage after surgical clipping and endovascular coiling: insights from a large cohort study
Source: Front Neurol. 2025 Sep 10;16:1622819. doi: 10.3389/fneur.2025.1622819 (PMC12459112; doi:10.3389/fneur.2025.1622819)
Supplement: Supplementary file 1 [file Table_1.DOCX]

Supplemental Table 1 Baseline characteristics between included and excluded patients.

| Variables | Included (n = 467) | Excluded (n = 801) | *P* |
| --- | --- | --- | --- |
|  |  |  |  |
| Age, years | 56.00 (48.00, 64.00) | 55.00 (48.00, 63.00) | 0.311 |
| Max diameter of aneurysm, mm | 5.54 (4.00, 8.00) | 5.63 (4.00, 8.00) | 0.965 |
| Graeb score | 1.00 (0.00, 2.00) | 1.00 (0.00, 2.00) | 0.132 |
| GCS score | 14.00 (13.00, 15.00) | 15.00 (11.00, 15.00) | 0.977 |
| SEBES score | 2.00 (1.00, 4.00) | 2.00 (1.00, 4.00) | 0.249 |
| mFS Score | 3.00 (2.00, 4.00) | 3.00 (2.00, 4.00) | 0.391 |
| WFNS score | 2.00 (1.00, 3.00) | 1.00 (1.00, 3.00) | 0.359 |
| Hunt Hess score, M (Q₁, Q₃) | 2.00 (2.00, 3.00) | 2.00 (2.00, 3.00) | 0.074 |
| Gender |  |  | 0.757 |
| Female | 277 (59.31) | 468 (58.43) |  |
| Male | 190 (40.69) | 333 (41.57) |  |
| Treatment Modality, n (%) |  |  | 0.710 |
| Endovascular treatment | 213 (45.61) | 374 (46.69) |  |
| Surgical clipping | 254 (54.39) | 427 (53.31) |  |
| Smoking, n (%) | 80 (17.13) | 189 (23.60) | **0.011** |
| Drinking, n (%) | 51 (10.92) | 170 (21.22) | **<.001** |
| Diabetes, n (%) | 26 (5.57) | 53 (6.62) | 0.456 |
| Hypertension, n (%) | 245 (52.46) | 440 (54.93) | 0.395 |
| History Of Heart Disease, n (%) | 66 (14.13) | 151 (18.85) | **0.031** |
| History Of Antiplatelet, n (%) | 2 (0.43) | 5 (0.62) | 0.951 |
| History Of Anticoagulant, n (%) | 13 (2.78) | 36 (4.49) | 0.127 |

mFS: modified Fisher scale; SEBES: Subarachnoid Hemorrhage Early Brain Edema Score; IVH: intraventricular hemorrhage; GCS: Glasgow coma score; WFNS: World Federation of Neurological Societies

Table 2 The association between baseline TyG-BMI level and the risk of unfavorable outcome

|  | The number of events (unfavorable outcome) | Model 1 | | Model 2 | | Model 3 | |
| --- | --- | --- | --- | --- | --- | --- | --- |
|  |  | OR (95% CI) | P | OR (95% CI) | P | OR (95% CI) | P |
| All patients | 103 | 1.003 (0.997-1.010) | 0.302 | 1.011 (0.999-1.023) | 0.063 | 1.010 (0.998-1.023) | 0.088 |
| TyG-BMI |  |  |  |  |  |  |  |
| Q1 (<193.313) | 26 | 1.0(Ref) |  | 1.0(Ref) |  | 1.0(Ref) |  |
| Q2 (193.313-212.282) | 19 | 0.769 (0.387-1.531) | 0.455 | 1.699 (0.494-5.835) | 0.400 | 1.358 (0.338-5.456) | 0.667 |
| Q3 (>212.282-238.041) | 27 | 1.110 (0.585-2.109) | 0.749 | 2.942 (0.959-9.025) | 0.059 | 1.921 (0.535-6.896) | 0.317 |
| Q4 (>238.041) | 31 | 1.443 (0.767-2.713) | 0.255 | 3.942 (1.224-12.697) | 0.022 | 3.539 (0.988-12.681) | 0.152 |

Crude model: age, gender.

Minimally adjusted model: age, gender, Grabe, SEBES, IVH, GCS, WFNS, Hunt Hess score, loss of consciousness, treatment modality, hypertension, history of heart disease, postoperative ventriculomegaly, abnormal liver function, anemia, pneumonia, and DVT.

Fully adjusted model: age, gender, Grabe, SEBES, IVH, GCS, WFNS, Hunt Hess score, loss of consciousness, treatment modality, hypertension, history of heart disease, postoperative ventriculomegaly, abnormal liver function, anemia, pneumonia, DVT, max diameter of aneurysm, preoperative Glu, preoperative urea, preoperative eGFR, preoperative AST, preoperative ALB, preoperative CHO, preoperative CKMb, preoperative WBC, preoperative MONO, preoperative NEUT, and preoperative HGB.
